# The time-place maze: A novel behavioral apparatus for assessing integrated spatial-temporal processing in rats

**DOI:** 10.1371/journal.pone.0340114

**Published:** 2026-02-13

**Authors:** Fazel Isapanah Amlashi, Ali Ashkbari, Mehrdad Jahanshahi, Ahmad Sohrabi, Siavash Ahmadi-Noorbakhsh

**Affiliations:** 1 Neuroscience Research Center, Golestan University of Medical Sciences, Gorgan, Iran; 2 Neuroscience Research Center, Biomedical Research Institute, Golestan University of Medical Sciences, Gorgan, Iran; 3 Cancer control research center, Cancer control foundation, Iran University of medical sciences, Tehran, Iran; 4 Comparative Medicine Group, Preclinical Core Facility, Tehran University of Medical Sciences, Tehran, Iran; 5 National Committee for Ethics in Biomedical Research, Ministry of Health and Medical Education, Tehran, Iran; Northwestern University Feinberg School of Medicine, UNITED STATES OF AMERICA

## Abstract

Behavioral paradigms used to study hippocampal place and time cells typically focus on the perception of either space or time in isolation, rather than the interaction between these two dimensions. To address this gap, we developed the Time-Place Maze (TPM)s, a novel behavioral apparatus designed to require rats to integrate their perception of both spatial and temporal cues to obtain a reward. The maze consists of a start box (it consist Time-door), a reward box, and two connecting bridges of differing lengths. At the beginning of the task, the time-door opens with one of two possible delays: either 0 seconds or 3 seconds. In The short delay (0s) the short path is open to reward, and in the longer delay (3s) the long path is open. The TPM protocol includes three sequential phases: training, screening, and the main experimental phase. During the training phase, rats underwent four daily sessions. In the final phase, each rat completed 100 randomized tests designed to assess integration of time and place information in decision-making. Six adults male Wistar rats (weighing 230 ± 20 g) were included in the training phase; four met the screening criteria and progressed to the main phase. On average, rats required 16.25 corrective interventions during training. In the main phase, success rates across 100 trials ranged from 73% to 82%. Importantly, the total number of corrections in the main phase was significantly associated with overall task success (p = 0.019). These findings support the feasibility and utility of the TPM in animal models. The task provides unique opportunities in behavioral neuroscience research.

## 1. Introduction

Scoville and Milner’s 1957 findings on the role of the hippocampus in memory initiated a major shift in neuroscience research [1]. Pioneer studies on the cellular basis of hippocampal function in memory led to the discovery of place cells and time cells in the hippocampus [2,3].

Place cells, first identified by O’Keefe and Dostrovsky in 1971, were shown to fire at specific spatial locations during maze navigation in rodents, supporting the notion that the hippocampus constructs a cognitive map of the environment and encodes the spatial location of salient events [4–7]. The role of the hippocampus is not limited to spatial coding. The discovery of time cells by MacDonald et al. in the CA1 region introduced a new dimension — temporal coding — into the functional landscape of the hippocampus [8]. Time cells resemble place cells but are temporally tuned, firing in response to specific time intervals and contributing to the encoding of event sequences over time. Following their discovery, various behavioral challenges were developed to examine the function of time cells. For example, Kraus et al. used a figure-eight maze combined with a treadmill to investigate how time cells encode sequences of events and their interrelations [9]. Also, other challenges, such as odors sequence memory tasks, reveal their role in the chronological order of events in memory and time coding [10,11]. The presence of these cells in the CA1 and CA3 regions of the hippocampus has attracted the attention of scientists to these areas and their network interactions [7,9,12]. Recent investigations reported the presence of new and similar cells in the hippocampus that respond to degrees of time and distance simultaneously [9], suggesting the existence of neural mechanisms underlying integrated spatiotemporal perception. Supporting evidence from human fMRI studies, particularly in virtual reality navigation tasks, indicates that temporal and spatial information is processed within overlapping hippocampal networks [12].

Despite substantial progress, most existing behavioral challenges examine spatial and temporal perception independently, without addressing their integration. However, to investigate the relationship between time cells and place cells, a new challenge is needed in which the rodent integrates the perception of place and time to solve the challenge. To address this gap, we developed the Time-Place Maze (TPM), a novel behavioral challenge designed to assess integrated spatiotemporal cognition in rodents. In TPM an external time-delay cue (0s vs 3s) links to two alternative spatial routes with hidden exits, that are physically present and visually indistinguishable at the start. This study presents the design rationale, experimental protocol, and preliminary validation results of the TPM.

## 2. Materials and methods

### 2.1. Study design

The present experimental study was conducted between August 2020 and July 2021. Six adults male Wistar rats (weighing> 230 ± 20 g) were used in the experiment to evaluate the efficacy of the maze and its protocol. Animals were housed in the Golestan University of Medical Sciences (GOUMS) under standard environmental conditions. They were maintained at a constant temperature (22 ± 3ºC) with a 12/12 light/dark cycle, and they had free access to normal food and water. No physical or invasive procedures were performed on the animals; only behavioral observations were conducted. This study’s procedures followed the Guideline for the Care and Use of Laboratory Animals in Iran [13]. The bioethics committee of GOUMS approved the present study (ID: IR.GOUMS.REC.1399.181).

### 2.2. The maze apparatus

**Fig 1:**
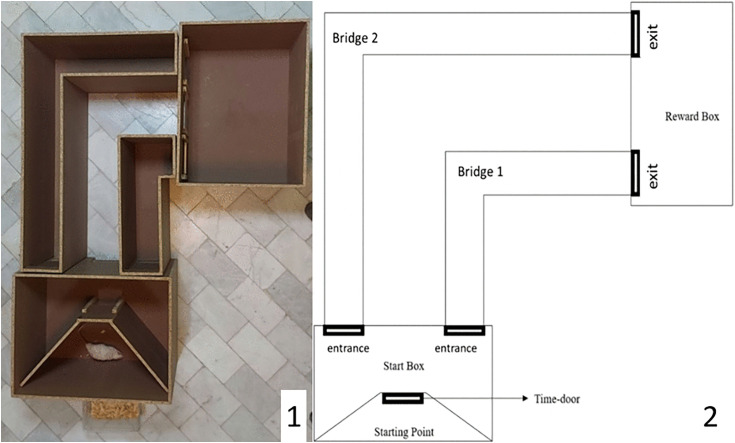
Image (1) and sketch (2) of the time-place maze that was used in the experiment. The maze apparatus includes: start box (length: 65 cm; width:60 cm; height: 60 cm), bridge 1 (longer arm: 40 cm; shorter arm: 15 cm, height: 60 cm), bridge 2 (longer arm: 90 cm; shorter arm: 55 cm, height: 60 cm), and reward box (length: 65 cm; width:60 cm; height: 60 cm). The starting point and time-door is located in the start box.

The TPM consists of four major parts connected to each other (Fig 1). The first part is the start box (length: 65 cm; width:60 cm; height: 60 cm); in the start box, there is a restricted area (starting point) with a guillotine-like door (time-door) at the front wall of it. The starting point is a place where an animal should be placed for starting the test and it keeps the animal focused on the time-door. The shape of the Time-door is the same as that of other guillotine-like doors that are used in T-maze or Y-maze. We redefined its concept by adding a delay in opening time to entangle the concept ot time to the very first object and variable of the maze. This door opens at specific times after placing the rat in the maze, and its function is described in the next part of the article.

The second part is the reward box (length: 65 cm; width:60 cm; height: 60 cm) which is similar to the start box, but without any restricted area. The reward for each test should be placed in the middle of this box.

The third part of the maze is bridge 1 (longer arm: 40 cm; shorter arm: 15 cm, height: 60 cm) which connects the start box to the reward box. This bridge has guillotine doors at the beginning (entrance) and the end (exit). Bridge 1 is the shorter path to the reward box.

The fourth part is bridge 2 (longer arm: 90 cm; shorter arm: 55 cm, height: 60 cm) which has the same features as bridge 1 but is substantially longer. Both bridges are L-shaped. The angled leg of the bridge hides the status of the exits when a rat is at the beginning of the bridge; thus, the rats could not visually decide which bridge to take.

### 2.3. Maze design rationale

The TPM is designed to provide a behavioral challenge depending on the perception of the time and place of an event. Therefore, there are two kinds of tests in the TPM protocol that contains temporal and spatial factors:

I Bridge 1 test: At the beginning, the entrances of both bridges are open, and only the exit of bridge 1 is open. After placing the rat in the starting point, the time-door opens without any delay (0 second).
II Bridge 2 test: At the beginning, the entrances of both bridges are open, and only the exit of the bridge 2 is open. After placing the rat in the starting point, the time-door opens after a 3s delay.

In order to solve these tests, the rat should figure out two relationships between the opening time of the time-door and the bridges:

I Whenever the time-door opens immediately (short opening time), only the exit of bridge 1 (short path) is open to reaching the reward.
II Whenever the time-door opens with delay (long opening time), only the exit of bridge 2 (long path) is open to reaching the reward.

The challenge of the TPM can be solved by merging the temporal data with spatial information as described above. We hypothesized that if a rat becomes successful in a certain occasions of the bridge 1 and 2 tests, it can be concluded that the rat has learned the logic of the TPM.

### 2.4. Protocol

**Fig 2:**
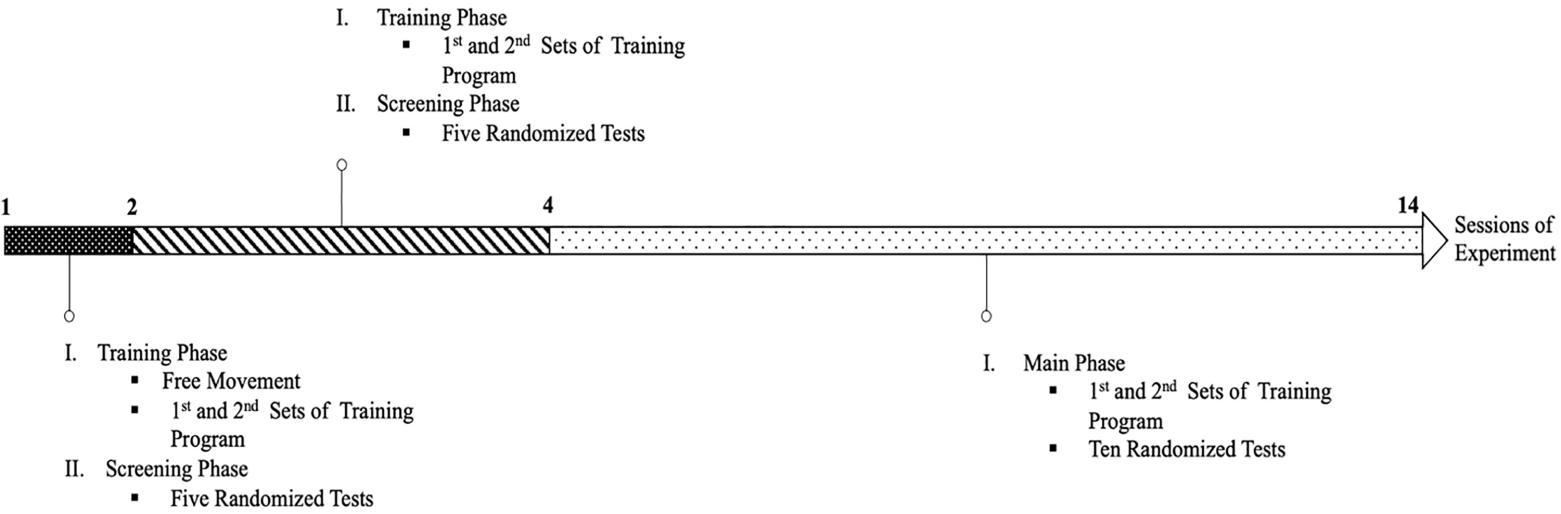
Time line of the time-place maze protocol according to the sessions of each phase. The experiment was done in three phases included training phase, screening phase, and main phase.

The goal of the TPM protocol is to guide training, screening, and implementation of the tests. It is designed based on the protocol of previous similar mazes [14–16]. The overview of the protocol is presented as a timeline according to the experiment sessions (Fig 2). The protocol includes several phases. Each phase consists of multiple sessions, and each session follows a specific program.

Rats were feed-restricted 24 hours before each session of the experiment with respect to maintaining 80% of their initial body weight to improve their tendency to explore [17,18]. For this purpose, the rats were weighted at the beginning of the experiment. Then every rat weighted before each feed-restriction and before each session of the protocol in order to exclude the rats with more than 20% weight loss compared to their initial weight. Water was provided to rats ad libitum. The feta cheese was used as the reward in this experiment.

#### 2.4.1. Training phase.

**Fig 3:**
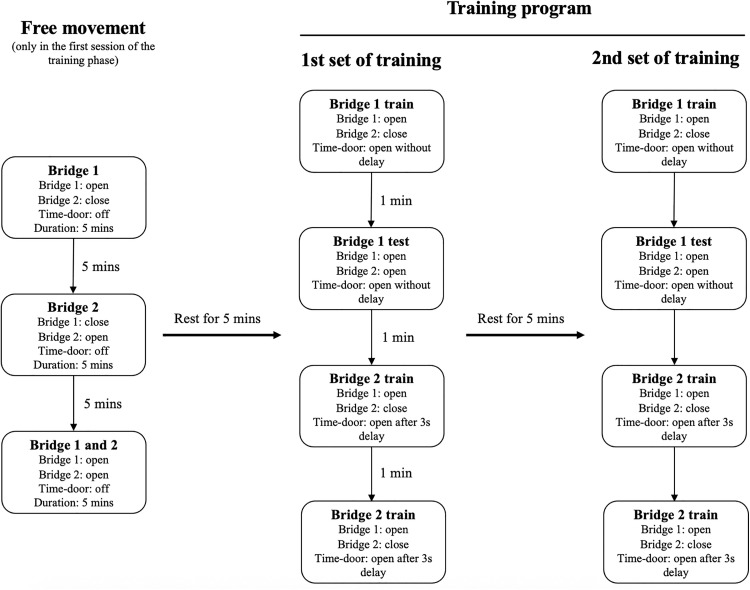
Details of a session of the training phase in the time-place maze protocol. Free movement was only implemented in the first session of the training phase. Training program (1st and 2nd sets of training) was implemented in all sessions of the training phase and main phase of the experiment.

The training phase is the first part of the protocol. This phase consists of four training sessions with the same program repeated daily (Fig 3).

Before starting the training program in the first session, give time to rats for free movement in the maze to reduce the stress and familiarize them with the space. we suggest to organize this free movement into three parts; let the rat explore the maze in each part for 5 minutes and then rest for 5 minutes in the cage until the next part (Fig 3) (Supplementary video 1):

I Bridge 1: Keep all maze doors open except for the entrance and exit of Bridge 2, which should be completely closed to prevent access.
II Bridge 2: Keep all maze doors open except for the entrance and exit of Bridge 1, which should be completely closed to prevent access.
III Bridge1 and 2: All doors are open.

The training program includes two sets of tests similar to each other and should be implemented with a 5–10 minutes rest between them (Fig 3). Each set consists of four consecutive tests (supplementary video 2):

I Bridge 1 train: The entrance and exit of bridge 1 are open; both doors of bridge 2 are closed. After placing the rat at the starting point, the time-door opens at the same time without delay.
II Bridge 1 test: At the beginning of the session, the entrances of both bridges are open, but only the exit of Bridge 1 remains open. After placing the rat at the starting point, the time-door opens immediately, with no delay.
III Bridge 2 train: The entrance and exit of Bridge 2 are open, while both the entrance and exit of Bridge 1 remain closed. After placing the rat at the starting point, the time-door opens following a 3-second delay.
IV Bridge 2 test: At the beginning, the entrances of both bridges are open, and only the exit of the bridge 2 is open. After placing the rat at the starting point, the time-door opens following a 3-second delay.

These four tests show the logic of the maze to a rat. The bridge trains help the rat to understand that the short and long opening time of the time-door is associated with the shorter and longer paths, respectively. Bridge 1 and 2 tests train the rat to choose a path based on the association that was showed in the previous single bridge test. Overall, this training program prepares the rat for randomized tests in the main phase.

#### 2.4.2. Correction.

In the training phase, rats may fail individual tests. In such cases, appropriate corrective feedback should be provided to facilitate learning. Positive correction is particularly important in early training, as the complexity of the maze often results in a high error rate during initial sessions.

**Fig 4:**
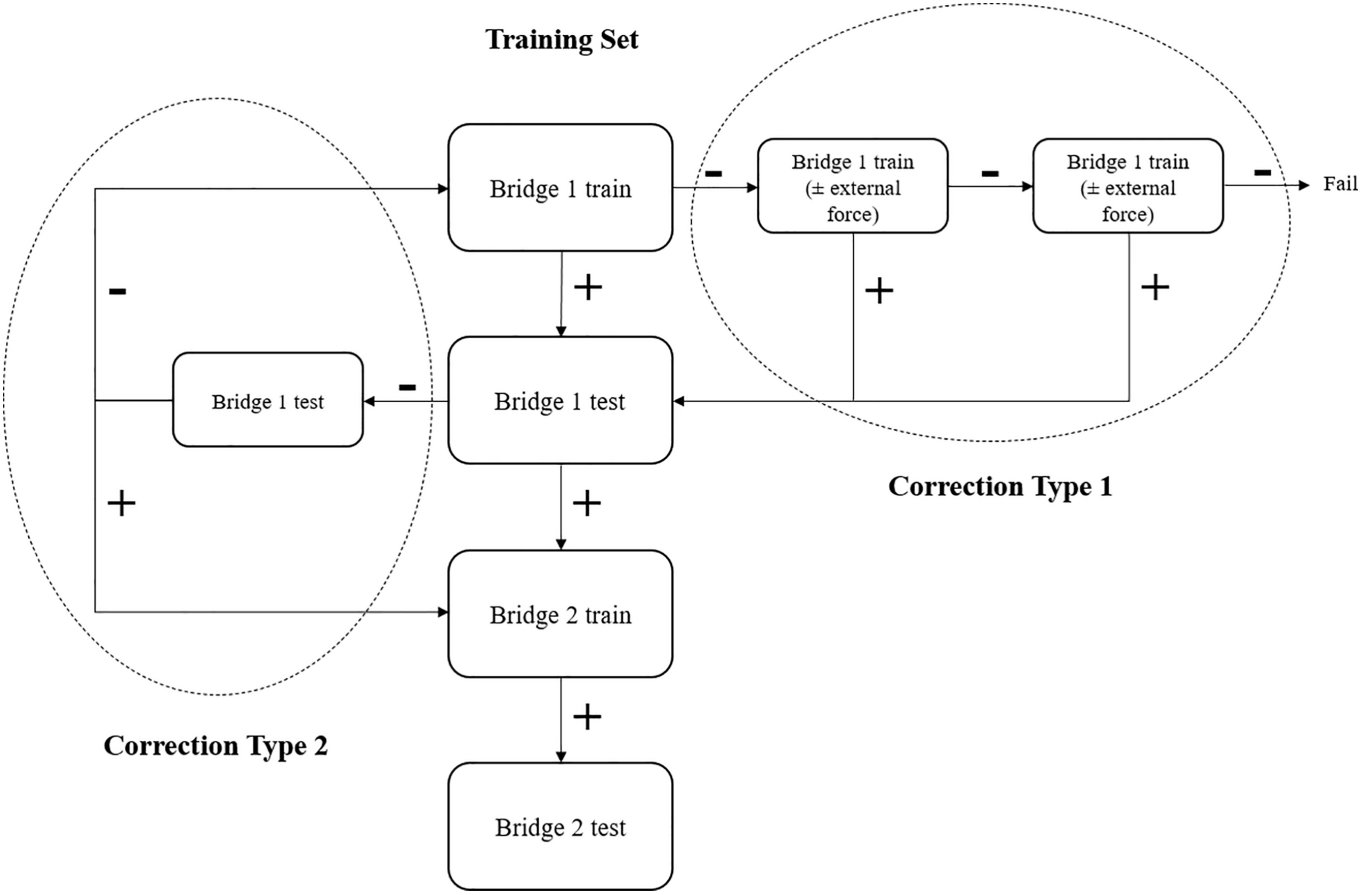
Algorithmic approach to correcting the failure of a rat in the training program of time-place maze protocol. The correction type 1 was used in bridge trains failure, and the correction type 2 in bridge tests failure.

There are two types of potential failures in the training phase: (1) failure during bridge training, and (2) failure during bridge testing. To simplify the correction procedure, an algorithmic approach is employed (Fig 4). Correction Types 1 and 2 correspond to failures in bridge training and bridge testing, respectively.

If a rat fails the same test more than three consecutive times, it should be excluded from that day’s training (a “failed day”) and the session should be repeated on another day. Rats accumulating three or more failed days during the training phase are recommended to be excluded from the experiment, because it seems that they are not potential to continue the protocol.

#### 2.4.3. Screening phase.

**Fig 5:**
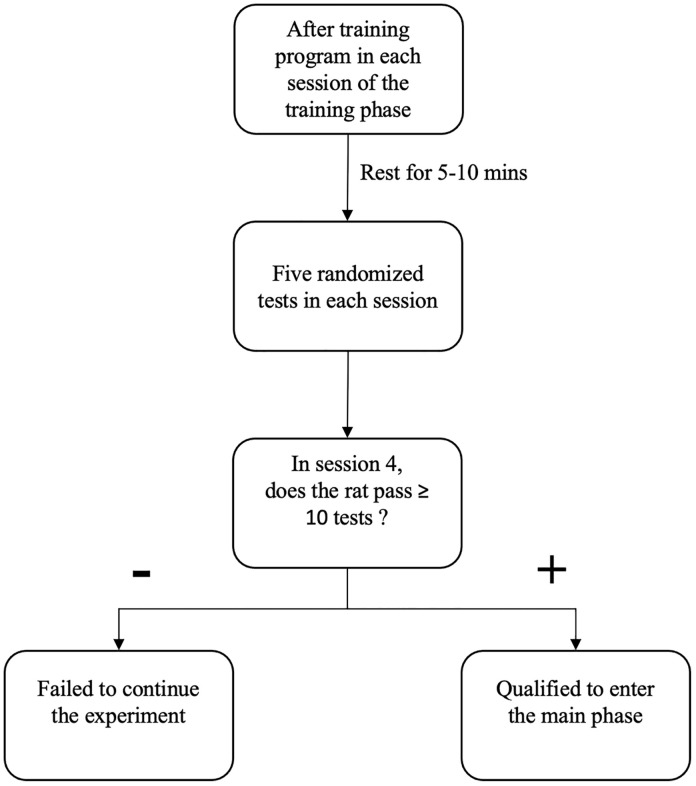
Details of screening phase in the time-place maze protocol.

The screening phase was conducted over four consecutive days, during which each rat completed a total of five randomized tests per day (Fig 5). This multi-day screening approach reduces the likelihood of chance performance compared to single-day screening and provides a more reliable measure of overall task acquisition. It also minimizes experimental waste in terms of time, resources, and animal effort. Rats that successfully completed at least 50% of the 20 total screening trials were enrolled in the main phase of the protocol.

#### 2.4.4. Main phase.

The main phase is the last part of the protocol and is also a critical point of the experiment because the results of this phase support or reject the hypothesis of the maze. The tests of this phase show whether the rat learns the logic of the maze or not. If a rat successfully passes more than 70% of tests, it can be concluded that the rat understands the logic of the maze and shows a kind of behavior that is highly dependent on the merging of the temporal and spatial perceptions.

**Fig 6:**
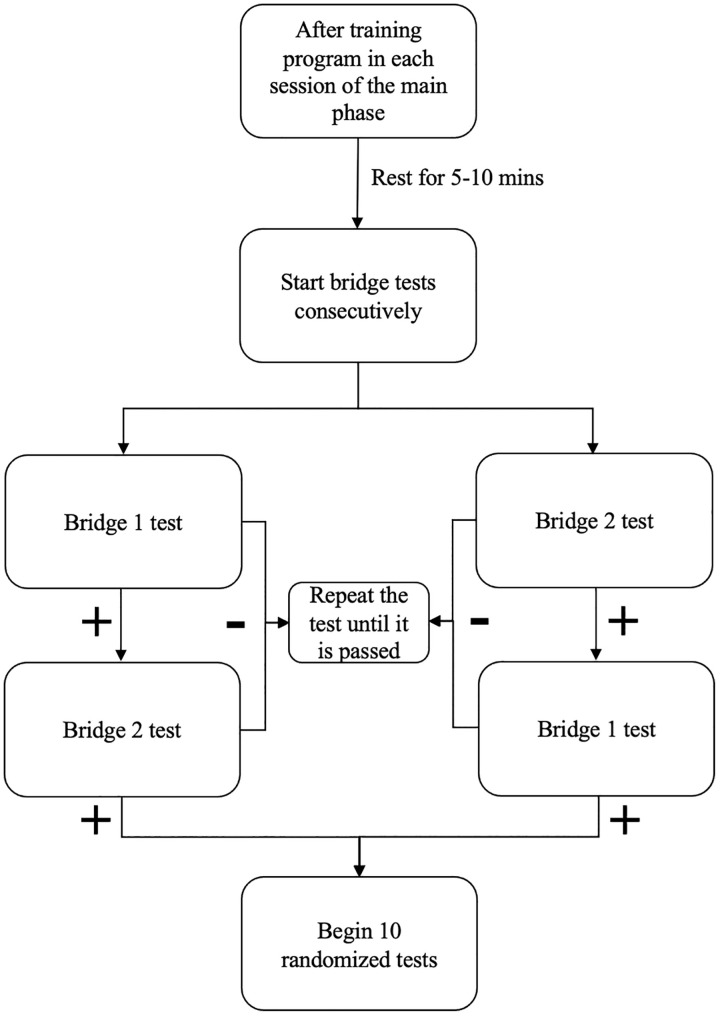
Algorithmic approach to implementation of the main phase in the time-place maze protocol.

The main phase was implemented for ten sessions in this study and its length is optional for future studies (Fig 6). First, the training program (with correction) should be conducted each day. The necessity of training on each day of the main phase is to boost the learning of the rat because the challenge of the maze is complicated and on the other hand, this challenge is not about the spatial memory but it is about the presentation of a real-time behavior with high involvement of brain analysis. Second, consider 5–10 minutes for rest after the training program and then the operator gives consecutive bridge tests to the rat until it becomes successful in one bridge 1 and one bridge 2 tests consecutively (no matter which tests comes first) (Fig 6). Then, consider the 60s rest for the rat and start ten randomized tests immediately (the principle of randomization is described below) (Fig 6) (Supplementary video 3).

Exclusion Criteria During the Main Phase:

- If the rat repeatedly chooses only one bridge (e.g., always selects Bridge 1).
- If the rat fails to choose a bridge in more than three consecutive tests.
- If the rat remains stationary, showing no movement or doesn’t choose any doors during three consecutive tests.

In either case, the session should be terminated and rescheduled for a later day, with the entire session repeated from the beginning.

#### 2.4.5. General consideration for the trains and tests.

The following points were considered in all bridge trains and tests in every phase of the protocol:

I Acclimatize the rat to the experimental environment by placing it in its cage for 5–10 minutes before each series of tests or train.
II Put a reward in the reward box for all trains and tests in training, screening and main phases.
III When a rat is placed at the starting point, a short loud sound (60 dB and 2000 Hz) like an alarm should be played for 1s to show that the test or train begins.
IV When a rat reaches the reward box, the train or test should be considered successful; if a rat chooses the wrong bridge, the rat is failed. In both cases, the rat should be returned to its home cage at the end of the trial.
V The maximum time for solving the test or train is 90 seconds; if a rat did not choose any bridge at this time, the test is failed.
VI The resting period between trains or tests is 60 seconds.
VII Prepare maze for the next test or train in resting period between them. The preparation for each test includes: cleaning the maze (remove food shavings, stool, and etc.), putting reward for the next test, and checking the doors.
VIII To minimize the operator’s presence effect, the maze’s high walls limit the rat’s view, leaving only the reward box visible during the experiment.
IX In the case of operator’s faults in every part of the protocol, that part should be paused and begins again after 5–10 minutes to prevent any adverse effects on the training of the rat.

#### 2.4.6. General consideration for randomization of the tests.

For randomizing the order of the tests in the screening or main phases of the protocol, Randomics–Rnd Generator Full V 1.7 application was used in the study in order to reduce bias in determining the combination of bridge tests. In addition, the following items should be considered:

I In the tests’ sequence of the main phase session, the maximum number of the bridge 1 or 2 tests that can be repeated in a row is three times, because more than this number may become a cause for bias toward a bridge in a rat.
II It is suggested to dedicate the first tests of sequences equally to each bridge test.
III The number of the bridge 1 and bridge 2 tests in a sequence should be equal; in other words, there should be five bridge 1 and 2 tests among ten tests of the main phase session.
IV In 20 tests of the screening phase, also the number of the bridge 1 and bridge 2 tests should be equal.

### 2.5. Statistical analysis

Operators filled out a checklist with complete details during each session of the experiment to keep all the information organized.

Statistical analyses of the obtained data were performed using R software (version 4.2.0).

Data visualization was conducted with the “ggplot2” package, and estimated marginal means were obtained using “least squares means” to compare rats’ performance trends over sessions. The Tukey correction was used for adjusting the Pairwise comparisons for multiple testing.

To account for correlated measurements within rats, Generalized Estimating Equations (GEE) were implemented using the “geepack” package, specifying Rat as the clustering variable. Models assumed a Gaussian family with an identity link function, and three correlation structures (independent, exchangeable, AR (1)) were compared using QIC to select the optimal fit. Parameter estimates (β ± SE) were evaluated using Wald χ² tests with robust standard errors, and significance was defined as p < 0.05 (two-tailed).

## 3. Results

### 3.1. Training phase

Overall, six rats were entered into this experiment. Four rats successfully completed their four-session training program without any failed day; the other two failed in three days of the training phase and were excluded from the experiment.

The details of findings in this phase are presented in Table 1. The mean number of corrections during the training program was 16.25 times per rat and more for the bridge 2 test than the other (63.1%). rats passed the bridge trains with fewer mistakes than bridge tests significantly. The correction was used for rat 3 more than the other rats with 22 times; whereas, rat 4 with ten times had the lowest rate. None of these four rats experienced a failed day in the phase.

**Table 1:** Results of the training and screening phases of the time-place maze.

			Rat 1	Rat 2	Rat 3	Rat 4
Training phase						
	Correction, N (%)					
		Bridge 1 train	0 (0%)	0 (0%)	0 (0%)	0 (0%)
		Bridge 1 test	6 (33.3%)	7 (46.6%)	5 (22.7%)	5 (50%)
		Bridge 2 train	0 (0%)	1 (6.7%)	0 (0%)	0 (0%)
		Bridge 2 test	12 (66.6)	7 (46.6%)	17 (77.3%)	5 (50%)
		Total	18 (100%)	15 (100%)	22 (100%)	10 (100%)
	Mean of correction per day		4.5	3.75	5.75	2.5
	Failed days, N		0	0	0	0
	Sets without mistakes, N		2	1	1	1
Screening phase						
	Total successful bridge tests, N (%)		13 (65%)	14 (70%)	13 (65%)	17 (85)
	Successful bridge 1 tests, N		8 (61.5%)	7 (50%)	6 (46.2%)	9 (53%)
	Successful bridge 2 tests, N		5 (38.5%)	7 (50%)	7 (53.8%)	8 (47%)

### 3.2. Screening program

Randomized tests of the screening phase for each day were presented in Table 2. There were an equal number of bridge 1 and 2 tests over four sessions.

**Table 2:** Sequence of the randomized tests in the screening and main phases of the time-place maze.

	Session	Sequence of tests
Screening phase	1^st^	1-2-1-2-1
	2^nd^	2-1-2-2-1
	3^rd^	1-2-2-1-1
	4^th^	2-1-1-2-2
Main phase	1^st^	2-1-2-1-2-1-1-1-2-2
	2^nd^	1-2-1-2-1-1-2-2-1-2
	3^rd^	2-1-2-1-2-1-2-1-1-2
	4^th^	1-2-1-2-2-1-2-2-1-1
	5^th^	2-1-2-1-2-1-2-1-1-2
	6^th^	1-2-1-2-1-2-2-1-2-1
	7^th^	2-1-2-1-2-2-1-1-1-2
	8^th^	1-2-2-1-1-2-2-1-1-2
	9^th^	2-1-2-1-1-2-2-1-2-1
	10^th^	1-2-1-2-1-1-2-2-2-1

In the screening phase, all the rats passed at least 60% of the tests successfully and were qualified to enroll into the main phase of the experiment (Table 1). Overall, the rats were more successful in the bridge 1 test than in the bridge 2 test (85% vs. 68%; p-value = 0.00; Table 1). Rat 4 had the highest success rate, solving 85% of the tests. The rats with lower needs for correction approach in the training phase, such as rats 2 and 4, had a higher success rate in the screening phase.

### 3.3. Main phase

Ten sequences of randomized tests were selected for ten sessions of the main phase (Table 2). In each sequence, there were equal numbers of bridge 1 and 2 tests. Also, none of the bridge tests were repeated more than three times in a row among days of the phase.

The mean of correction in 10 days of the main phase was 30 times for each rat; the lowest and the highest number were for rats 1 and 3, respectively (17 vs. 40). The details of findings in the training program of the main phase are showed in Table 3. Overall, the correction approach was used more in the first set of the training program than in the second one (69 vs. 61). Same as the training phase, the need for correction in the bridge 2 test (64.1%) was more than in the other tests. None of the rats had a failed day in the training program of the main phase.

**Table 3:** Results of the training program in the main phase of the time-place maze.

				Rat 1	Rat 2	Rat 3	Rat 4
Training program							
	Correction, N (%)						
		1^st^ Set					
			Bridge 1 train	2 (11.8%)	1 (7.7%)	0 (0%)	1 (5%)
			Bridge 1 test	6 (35.3%)	6 (46.15%)	4 (21%)	6 (30%)
			Bridge 2 train	2 (11.8%)	0 (0%)	0 (0%)	1 (5%)
			Bridge 2 test	7 (41.2%)	6 (46.15%)	15 (79%)	12 (60%)
			Total	17 (100%)	13 (100%)	19 (100%)	20 (100%)
			Set without mistakes	4	1	4	4
		2^nd^ Set					
			Bridge 1 train	2 (20%)	1 (9.1%)	0 (0%)	0 (0%)
			Bridge 1 test	2 (20%)	3 (27.3%)	4 (19.05%)	7 (36.85)
			Bridge 2 train	2 (20%)	0 (0%)	1 (4.7%)	2 (10.6)
			Bridge 2 test	4 (40%)	7 (63.6%)	16 (76.25)	10 (52.6%)
			Total	10 (100%)	11 (100%)	21 (100%)	19 (100%)
			Set without mistakes	3	3	3	2
		Total		27	24	40	39

All rats in the main phase passed at least 70% of randomized tests in 10 sessions (Table 4). Rat 3 and 4 scored the highest success rates among the other rats. The GEE model showed that the total number of corrections in the main phases had a significant relationship with the success rate in the main phase (p-value = 0.019); whereas the number of total corrections in the training phase (p-value = 0.75) and the success rate in screening phase (p-value = 0.49) did not have a significant association with it.

**Table 4:** Findings of the 100 randomized tests in the main phase of the time-place maze.

	Rat 1	Rat 2	Rat 3	Rat 4
Successful bridge 1 tests, N (%)	39 (53.4%)	42 (59.1%)	44 (53.6%)	45(56.2%)
Successful bridge 2 tests, N (%)	34 (46.6%)	29 (40.9%%)	38 (46.4%)	35 (43.8)
Total Successful tests	73 (100%)	71 (100%)	82 (100%)	80 (100%)
Regression line slope (CI 95%)	3.091 (0.45; 5.73)	− 0.545 (−3.19; 2.10)	2.667 (0.02; 5.31)	− 0.485 (−3.13; 2.16
Failed day, N	0	3	2	2

**Fig 7:**
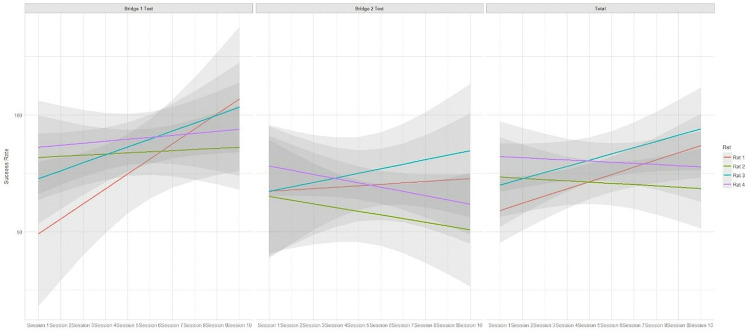
Trends of successful randomized tests in ten sessions of the main phase of time-place maze for each rat with respect to the bridge 1 and 2 tests.

During the sessions of the main phase, all the rats showed slight changes in the success rate (Fig 7); rats 1 and 3 had an increasing trend while rats 2 and 4 showed a declining trend in solving 100 randomized tests. All the rats showed a rising trend in success rate for the bridge 1 test but not for the bridge 2 tests. The trends and comparison of the trends with each other were not statistically significant.

All the rats were more successful in the bridge 1 test than in bridge 2 tests (170 VS. 136) (Table 4). The results of GEE model indicated that the total number of corrections in the training programs did not have any significant association with the success rate in one of the bridge tests. The GEE results were stable across different correlation structures, with the independence model showing the best fit according to QIC. Also, the success rate in the screening phase showed no relation with the success rate in bridge tests.

The difference between the success rate of doing bridge 1 and bridge 2 tests was 5–13 for the animals (Table 4). Rats 1 and 3 had the lowest difference, but rats 2 and 4 showed a larger difference in this regard. It seemed there is an association between the trend and balance in solving both bridge tests because the rats with a lower difference in solving bridge 1 and 2 tests showed an increasing trend than others (Fig 7).

## 4. Discussion

The present study aimed to design and establish a TPM for using in behavioral neuroscience. The range of success rate in randomized tests were 73–82% in the experiment. This rate can be accounted as primary proof of the understandability of the challenge for the animal models. Therefore, this behavioral challenge can potentially be used in future experiments studying time and place perception.

The success rates observed in the TPM were comparable to those reported in previous behavioral paradigms designed to study time and place cells. For instance, Mann et al. [19] reported a 78.6% success rate in an odor-sequence task, while MacDonald et al. [11] experiment, the challenge of object-delay-odor was implemented in order to study time cells. Four rats were enrolled in that experiment and the mean success rate was 77%, the same as our study. These similarities suggest that despite its complexity, the TPM yields consistent behavioral performance. Thus, TPM may serve as a reliable and efficient tool for future investigations of hippocampal function.

Rats exhibited two instinctive behaviors that conflicted with the intended logic of the TPM. The first was the tendency to explore; in this case, a rat preferred to choose a bridge different from its previous choice [16]. However, Yuki and colleagues demonstrated that information-seeking behavior, though instinctive, becomes increasingly adaptive as a result of training and learning [20]. Second, some rats relied solely on immediate feedback, repeating successful behaviors or avoiding failed ones without considering the time-path association. These two instinctive behaviors hindered logical decision-making, which is critically needed in solving our maze. Therefore, the training program was designed and used in every session to reduce the adverse effects of these forces and send positive feedback to the rats. The stability in the success rate of the rats during the main phase shows the protocol was somehow efficient in controlling the negative effects of instinctive behaviors and improving the logical reasoning in rats.

During the ten sessions of the main phase, two distinct patterns in performance trends were observed. Rats 1 and 3 with increasing patterns showed a balance in success rate between two bridges tests; while rats 2 and 4 significantly differed in the number of successful bridge 1 and 2 tests. The results showed an association between the trend in successful tests and the equilibrium in solving both bridge tests. Therefore, the rat that understands the logic of training for both bridges equally can improve its function in the main phase of the experiment.

All rats demonstrated higher success rates in the Bridge 1 tests, suggesting that Bridge 2 posed greater difficulty. We have found similar evidence in other reports. It is shown that rats could have a right- or left-side bias in behavioral challenges [21]. Also, in a study by Bak et al. [22], the rats showed spatial preferences in a Y-maze. It can be concluded that the bias toward a bridge in TPM was not unexpected and the spatial preferences of rats may be a reason for these results. In addition, the longer length and unfamiliarity of Bridge 2 may have introduced higher anxiety, leading rats to favor the shorter, more familiar path. These factors likely contributed to the observed performance differences.

There is another justification for this finding that may be more fundamental than the other and that is related to the logic of the maze. In this maze, the operators tried to induce causal reasoning in rats based on the concurrent elements (the time, the path, and the reward). The rat should understand that the shorter time links to the shorter path and the longer time links to the longer path. As bridge 1 is the short and fast way to reach the reward box, the process of casual reasoning for this bridge may seem more straightforward to figure out by the rat because the base of causal reasoning is the synchrony of the cause and effect. Prior studies also suggest a natural preference in rats for shorter, more direct paths to rewards [23]. Although all of these justifications for spatial preferences need to be noted but the results of this experiment showed that the constant training program could reduce the level of adverse effects on the reasoning of the rats.

Same as the other experiments, we faced some limitations in this study. Considering these limitations in future studies can improve the TPM for the research in this field. The first and the most significant limitation was the low number of rats in the experiment. In order to reach to more decisive results, the number of animals should be increased. Second, due to our financial and technical limitations, the maze’s doors used in this experiment was not automated. In future studies, we suggest to automate the doors of the maze especially using automated doors with remote control in order to reduce the presence and involvement of human operators in the room. Another important limitation of this study is the absence of cellular or neurophysiological data. As a result, all interpretations are based solely on behavioral performance, without direct evidence linking observed behaviors to underlying neuronal activity.

In conclusion, the findings of the experiment showed that the TPM was applicable in animal research and can be used for the evaluation of cognitive functions. Since the goals of the maze were more complicated compared to previous challenges, the complexity of its protocol is expected and we hope this issue will not be an obstacle in the way of future studies.

Three categories of studies are suggested to be conducted in the future based on the TPM. First, more experiments are needed to verify the applicability of the maze and the efficacy of its protocol. Second, further studies can aim to improve the structure and develop the protocol of the maze in order to make the TPM a better tool for research in this field. Third and the most important, TPM can open a new window into cognitive neuroscience research by letting the researcher study a cognitive behavior about the integrated perception of dimensions from a new view that was not available before. Therefore, future studies could focus on the investigations of the cellular, molecular, and other aspects of such behavior in order to expand our knowledge about brain functions and structure.
